# An Autoethnographic Account of Familial Mediterranean Fever: A Turkish Patient’s Discovery of Spiritual Meaning

**DOI:** 10.1007/s10943-025-02253-2

**Published:** 2025-01-27

**Authors:** Cigdem Yuksel

**Affiliations:** Psychiatric Nursing Department, Gulhane Faculty of Nursing, University of Health Sciences, Turkiye, 06010 Etlik, Ankara Turkey

**Keywords:** Autoethnography, Qualitative study, Spirituality, Chronic illness, Familial Mediterranean fever

## Abstract

Familial Mediterranean Fever (FMF) is a genetic autoinflammatory disorder characterized by recurrent febrile episodes that are accompanied by pain in the abdomen, chest, or joints caused by peritonitis, pleuritis, skin lesions, arthritis, and pericarditis. This original article aims to provide an analytic autoethnographic account of a Turkish patient’s experience of FMF, with a focus on the discovery of spiritual meaning. In addition to discussing the grief reactions to a loss of health, the article uses self-reflexive discourse and narrative-based analysis to explore four stages of discovery of spiritual meaning through FMF: “omnipotent me,” “God’s punishment,” “God’s test,” and “God’s mercy.” The article provides an in-depth look at the experience of FMF, a chronic and lifelong disease, through a spiritual lens and offers suggestions for mental health professionals and rheumatologists providing holistic treatment to FMF patients that might improve treatment adherence.

## Introduction

### Familial Mediterranean Fever

FMF is a genetic disorder characterized by recurrent febrile episodes that are accompanied by pain in the abdomen, chest, or joints caused by peritonitis, pleuritis, skin lesions, arthritis, and pericarditis (Ozdogan & Ugurlu, 2019; Tufan & Lachmann, 2020). Individuals with genetic origins in the Mediterranean basin, predominantly Sephardic Jews, North African Arabs, Armenians, Turks, Greeks, and Italians, are more frequently affected (Tufan & Lachmann, 2020). Diagnosis is largely clinical, although molecular genetic testing is available. FMF cannot be cured, but it can be controlled with the lifelong use of colchicine (Satiş et al., 2020; Yüksel et al., 2019). Almost every FMF patient experiences fatigue, diarrhea, abdominal pain or tenderness, and a “board-like” firmness of the abdominal wall (Wekell & Wester, 2022). Most of them experience severe joint pain and inflammation, which can be triggered by minor trauma or sustained exertion (Ozdogan & Ugurlu, 2019).

There are also common cardiovascular manifestations of FMF, like pericarditis, and pulmonary manifestations like painful breathing (Alsarah et al., 2017; Tsur et al., 2021). Some FMF patients may develop a serious condition known as amyloidosis, which can eventually progress to cause kidney failure (Ozdogan & Ugurlu, 2019; Tufan & Lachmann, 2020). Another symptom is infertility, which is caused by ovulatory dysfunction and peritoneal adhesions in women, and testicular failure in men. In pregnant FMF patients, an increased occurrence of miscarriage has been found (Atas et al., 2020).

Although episodes of FMF can occur spontaneously for no identifiable reason, certain triggers have been identified in some cases, such as infection, trauma, psychological stress, vigorous exercise, journeys with long duration in vehicles or frequent travel, exposure to cold, fat-rich food, the menstrual cycle, excessive physical activity, and certain drugs (Tufan & Lachmann, 2020). The amount of time between episodes varies and can range from a week to a few months, and most patients feel healthy between episodes, which last for about four days and go away without treatment (Wekell & Wester, 2022; Yüksel et al., 2019). The first episode usually occurs by the age of 20 years, but unfortunately, patients live with symptoms for nearly seven years before they are diagnosed with FMF (Yüksel et al., 2019). By that time, they had sought treatment for FMF symptoms that were associated with irrelevant diseases, underwent a large number of laboratory tests, used unrelated drugs, and had unnecessary surgeries until finally meeting with a rheumatologist and being diagnosed with FMF (Wekell & Wester, 2022; Yüksel et al., 2019).

The clinical burden of FMF has a significant impact on psychological, social and economic aspects of a patient’s life, leading to disability and poor quality of life (Kacan et al., 2023). Hausmann et al. (2018) reported that autoinflammatory diseases, including FMF, impact the physical, social and emotional aspects of patients and their families. They showed that feelings of uncertainty were a hallmark of families and patients not only before but also after the diagnosis, which was one of the most emotionally challenging factors (Hausmann et al., 2018). Uncertainty, sudden and unexpected onset of episodes, and loss of roles and functions contribute to psychological effects such as anxiety and depression (Kacan et al., 2023; Lidor et al., 2021; Yüksel et al., 2019). Lidor et al. (2021) showed that stress-related depression and anxiety in FMF patients is a vicious circle that also causes new attacks.

The loss of roles and functions has not only psychological but also social consequences (Erbis et al., 2018; Yüksel et al., 2019). In a phenomenological study conducted to explore life experiences and coping behaviors of Turkish FMF patients, Yüksel et al. (2019) showed that the physical effects of the illness, loss of social role and functions, unexpected FMF episodes, and stigmatization were the major concerns of patients. They mentioned that FMF symptoms affect everyday activities of the patients for almost 12–72 h and limit role performance like being a spouse, parent, employee, student, and related functions. Since the symptoms appear suddenly and regress within a few days, this situation viewed as unrealistic by the patients’ acquaintances (family, friend, employer, etc.) and causes stigmatization (Yüksel et al., 2019). In a study by Erbis et al. (2018) also mentioned that autoinflammatory diseases, including FMF, significantly affect physical abilities as well as social and psychological functions, including work productivity, employment, personal relationships, continuity of education and social activities.

Coping mechanisms to deal with everyday stressors are one of the main sources of skills in dealing with chronic diseases and their diverse effects on health and quality of life (Kristofferzon et al., 2018). To cope with the physical symptoms, patients follow carefully the prodromal symptoms of FMF, which consist of discomfort and psychological distress, and try to manage the circumstances that are considered triggering factors (dietary habits, physical conditions, fatigue, stressors, etc.) (Kacan et al., 2023; Yüksel et al., 2019). They also pay attention to medication adherence, and regular doctor visits (Satiş et al., 2020; Yüksel et al., 2019). To cope with psychological symptoms, FMF patients seek social or psychological support (Kacan et al., 2023; Lidor et al., 2021; Yüksel et al., 2019). Active support from family or friends during illness is an effective coping behavior and significantly improves the patients’ quality of life (Hausmann et al., 2018; Kristofferzon et al., 2018).

Spiritual/religious coping is another way to deal with chronic symptoms (Chen et al., 2016). Pargament defined religious coping as the search for meaning in times of stress, and mentioned that religion is present at every stage of the coping process, helping individuals discover meaning, gain control and acquire comfort through closeness to God, achieve closeness with others, and transform lives (Pargament, 2001; Pargament et al., 2000). Coping with health issues using spiritual/religious recourses is a widespread psychological mechanism common among physical patients. There are qualitative and quantitative studies in the literature on religious coping related with stressful events, infertility, pulmonary diseases, renal failure, and life satisfaction, which are also possible consequences of FMF, but so far there is only one qualitative and one quantitative study in the literature related to religious coping in FMF patients (Duran & Can Öz, 2023; Mesquita et al., 2022; Szafraniec et al., 2020; Yüksel et al., 2019; Şentürk et al., 2022). In the study on acceptance of illness and use of religious coping strategies conducted with Turkish FMF patients, Şentürk et al. (2022) showed that positive religious coping scores were higher than negative religious coping scores, and there was a weak, negative correlation between patients’ acceptance of the illness and their negative religious coping. The study also suggested that FMF patients tend to focus on their faith and religion to alleviate the problems they experience and adapt to the circumstances associated with their illness/treatment (Şentürk et al., 2022).

Although various studies show how spiritual people activate their meaning-making system when faced with illness, both qualitative and qualitative research on spiritual coping with FMF is scarce. This article aims to provide an analytic autoethnographic account of a Turkish patient’s experience of FMF, with a focus on the discovery of spiritual meaning. The results may shed light on the process of religious transformation and religious coping that is already available in the literature.

## Methods

### Autoethnography

Autoethnography is a qualitative research method that places the self at the center of cultural analysis in order to describe and systematically analyze the personal experience and to gain a better understanding of the connection between self and others (Hoque et al., 2017). Grounded in anthropology, this method is recognized as having the potential to develop new knowledge in the humanities, social sciences, and, increasingly, the health sciences (Bath, 2017; Peterson, 2015).

Autoethnography is the study of the interrelationships between self, society, context, and culture, as well as the use of personal experience to investigate the universality of those experiences in order to create descriptions of cultural experience to aid understanding (Adams et al., 2021; Turner, 2013). Autoethnography asks the researcher to engage in a reflexive, introspective process involving careful and rigorous thought to understand a situation by alternating between inward and outward lenses (Adams et al., 2017, 2021). This sociological introspection is the process of moving in and out of one’s thoughts and feelings about the experience to create effective autoethnographies (Adams et al., 2017).

Because the past is observed backward from the current moment, it is almost impossible to fully grasp experience; rather, the researcher’s goal should be to convey the meanings attached to the experience (Adams et al., 2017, 2021). However, the readers perceive the story from their own lenses, fed by their own experiences, and may not realize the real intent of the author (Adams et al., 2021; Koopman et al., 2020).

#### Using autoethnography in health research

While autoethnography has been little used and has not yet reached its full potential in the health disciplines, when used properly, it allows healthcare workers to tell their unheard stories as patients and invites other healthcare professionals to learn from their experiences and reflections. It also gives a chance to ensure a better understanding of illness experiences for individuals, families, and groups (Nowakowski & Sumerau, 2019; Peterson, 2015). Although autoethnography shifts the position of the healthcare researcher from objective observer to subjective participant, postmodern perspectives hold that there is no single correct form of knowledge, acknowledging and valuing multiple perspectives (Peterson, 2015; Wall, 2014). From the social constructivist view, the social world closely intertwines with the self, so subjective experience is inevitable and not only valid but also expected because individuals construct their sense of reality through interactions with others (Peterson, 2015; Pfadenhauer & Knoblauch, 2018).

#### Ethical Considerations in Autoethnographic Research

As an autoethnography describes an experience in a social context, it naturally includes interactions between self and others, which raises some ethical questions. Various views in the literature address ethical concerns in autoethnography. For instance, while some researchers have not considered it necessary to seek ethical approval for autoethnographic research because there are no research participants per se, some have offered to decide the ethical issues depending on whether the article is about the past or the present, whether the topic is potentially stigmatizing or controversial, and how prevalent others are in the text (Adams et al., 2021; Dahal & Luitel, 2022).

In addition, Turner (2013) suggests that while conducting autoethnographic research, the interaction between self and others still only represents the author’s own experience because “once words are spoken or actions performed; once they ‘leave’ the mouths or bodies of those who are part of the shared scenario in which the author was included, they become part of the author’s experience.” She mentions that “ownership” of these words and actions is passed out into the wider world to be interpreted and reinterpreted until they fade into dust. From that point of view, she questions needing to get permission from all the people who shared an experience, as well as whether obtaining such permission is even possible.

#### Ethical Considerations for this Autoethnographic Research

In this research, the source of data used in the autoethnographic account of experiencing FMF was obtained from a retrospective analysis of the author’s personal life experience. There are no research participants as outlined by research ethics committees, nor is there anyone identified as a conventional respondent for whom gaining informed consent is required. However, I informed all individuals mentioned in this autoethnography.

Analytic autoethnography is a unique method in which the researcher focuses the lens of inquiry on their own personal narratives in order to describe and systematically analyze the personal experience and to gain a better understanding of the connection between self and others (Hoque et al., 2017). In this analytic autoethnographic article, the author’s narrative about FMF with a focus on the discovery of spiritual meaning is discussed as four emergent themes. These themes, presented in the next section, emerged from systematic document analysis, which is the notes and diary of the author, and also self-reflexive discourse and narrative-based analysis.

## Findings and Discussion

### Prelude

*My housemate had decided to spend the weekend in another city. That meant two whole days at home alone. I was in the first year of my doctoral education, and I had a very intense academic program. I left work late on Friday night, but I still had a lot of things to do on the weekend—articles to read, and lecture presentations to prepare.*

*I stopped at the grocery store for a few items on my way home from work. I prepared my dinner quickly. I went into the living room with a tray in my hand and sat on the couch in front of the TV. I put on a movie I had rented. I realized that I was feeling tired. My body was exhausted. I was aching all over.*

*“I must be getting sick,” I said to myself. I hadn’t noticed until I got home, maybe because I was so focused on my studies. But I had been fine during the day*.

*I started watching the movie while I ate. By the time I got to the middle of the movie, it was almost impossible to eat anything. Every bite I swallowed caused terrible pain in my abdomen. Also, my body temperature was rising. I set the tray aside. I crawled back into the couch, curled up in the fetal position, pulled the sheet over my head, and started shivering with fever.*

*“I should get up and measure my temperature, or take a shower, maybe a fever reducer,” I reasoned. I was in so much pain that I could barely sit up. I wasn’t feeling well enough to stand up, and the pain in my abdomen was getting worse.*

*Still, I had to do something. “Come on, don't just sit around waiting,” I said to myself, and I tried to stand up by leaning on the edge of the couch. My God—the pain in my abdomen was so severe! I doubled over in agony on the couch. I stayed there for a while with my hand clutching my belly. Could it be food poisoning again?*

*Having these symptoms and ascribing them to food poisoning had been a recurrent experience for me for the last three years. The first experience was when I started to travel to another city, five hours away by bus, to obtain my master’s degree. My classes were on Tuesday and Thursday. I was at work the other five days in the city where I live. So I was on the road four times a week for two years, going and returning—a total of 80 h of travel each month. No pain, no gain.*

*Of course, I was exhausted during my journey; perhaps my immune system was compromised. Moreover, I did not eat homemade meals on the road. I ate in restaurants, and at least once a month, I suffered from stomachache, weakness, fatigue, fever, and diarrhea—all apparent signs of food poisoning. The symptoms lasted for one, sometimes three days, and then went away on their own.*

*But this was the first time my pain was so unbearable. Maybe it wasn’t food poisoning this time. “Oh no,” I thought, “do I have appendicitis?” Whenever I went to the emergency department with these complaints, they usually suspected appendicitis, kept me under observation for a while, and gave me an antipyretic. After making sure I didn’t have appendicitis, they finally would give me painkillers and send me home. I knew painkillers weren’t very helpful, though. I stopped thinking about my past: I had to go to the emergency department as soon as possible.*

*I called a friend, and she picked me up from the house, blankets wrapped around my body, and brought me to the hospital. Lying on the gurney in the emergency department was like rewatching a movie I had already seen over and over again. Same questions, same answers, same examinations, same results.*

*“I can’t say that painkillers will work very well if you prescribe them,” I told the doctor as he listened to my illness story. “During my last hospital admissions, the physicians even ordered Aldolan as the others did not help. But I’ve refused it lately because if it goes on like this, I am scared of becoming addicted.” I smiled weakly.*

*The doctor came closer to the gurney and pulled a chair up next to me. “This is happening more often than I thought, then,” he said*.

*“This year, every two to three months. But it was more frequent during my master’s degree, which lasted two years,” I said*.

*“Now, can you tell me what happened, starting from the earliest date you remember?” he said*.

*The emergency department was as crowded as always. With the sensitivity of a healthcare worker, I decided to explain it quickly, keeping in mind that patients with more urgent situations than mine might be waiting. I summarized my story. But the doctor continued to ask questions.*

*“Do you also have joint pain?”*

*“Joint pain? Right now, yes, my body is hurting all over. It’s probably related to my fever,” I said*.

*“No, I don't mean at the moment; think about it in general. Is there unilateral or bilateral swelling or redness of the joints?”*

*Out of the corner of my eye, I looked at his name, department, and rank on the nameplate on his doctor’s uniform. He was an emergency physician with the rank of captain*.

*“All my life? It’s happened, yes, but isn’t that normal? I'm also a commissioned officer like you, a paratrooper, and a diver. When I was doing heavy physical exercises as a part of my job, I had these symptoms. Don’t you experience the same?”*

*He smiled, nodded his head, and continued asking questions*.

*“Were there any exercises you had trouble maintaining because of pain in your joints?”*

*“Well, yes, I left the folk dance team. I felt a recurring pain in my foot. I was already very busy those days, and it was a social activity, not a necessity,” I said*.

*“I see. Do you have breathing difficulties or chest pain?”*

*I did not answer. I felt anxious. The ideas were like birds flying around in my head. I already had all that pain and fever. My teeth were chattering. They reminded me how these periods of fever and pain also affected my oral health. For those two- or three-day bouts, I preferred not to eat anything so that my abdominal pain would not increase. Moreover, fever and pain made it very difficult for me to maintain my oral hygiene. Most of the time, my gums felt itchy. On the third day of these symptoms, I would barely feel good enough to get out of bed to brush my teeth or eat.*

*“Should I mention this, too?” I thought. “Of course not; he isn’t a dentist. I already have an appointment with a dentist. He is not an orthopedist either, but he’s asking about bones and joints, too.”*

*“What about chest pain?” said the doctor again, looking carefully at my face*.

*“Now he is asking like a cardiologist,” I thought*.

*“You seem exhausted. Let's quit here and continue tomorrow,” he said*.

*“Tomorrow? I thought you would release me today.”*

*“No,” he said, “I am going to admit you to the hospital for tests. Also, I’ll have the rheumatologist visit you in the morning.”*

*The next morning, the doctor came back with a rheumatologist. My pain had eventually become moderate, and my fever was under control. I was curious why the rheumatology consultation was requested for the morning. The new doctor not only repeated the same questions but added many more as well.*

*“What are the triggers for the period of intense fever?”*

*“Food could be a trigger,” I explained.*

*“Does it coincide with menstrual periods?” he asked*.

*“Yes, from time to time.”*

*“What about exercise, insomnia, and periods of fatigue as triggers?”*

*“In my life, these are already routine; it coincides with these periods, yes,” I said*.

*“Is it possible to say that sometimes the symptoms start all of a sudden?” he asked with his eyebrows raised*.

*“Yeah, I was feeling pretty good during the day yesterday until I had pain.”*

*“Can you talk about the duration of your pain?” he asked while taking notes*.

*“My abdominal pain takes two or three days; my chest pain takes approximately a week. The joint pain sometimes lasts for a week or more, but I endure.”*

*“So, what do you do to cope?” he asked kindly.*

*I was silent for a while*.

*“I visited different specialists for fever, joint pain, abdominal pain, and chest pain when I needed to. I just used the medicine they prescribed.”*

*“Does anyone in your family have similar complaints?” asked the doctor*.

*“I have no idea.”*

*“Does anyone have kidney failure?” he asked*.

*“No, not as far as I know, but I’ll call my family and confirm. All these questions are starting to worry me. What do you suspect?” I asked anxiously*.

*“I see. We’ll need to do genetic testing, but your clinical findings suggest that it is Familial Mediterranean Fever, or FMF. It’s a chronic, genetic autoinflammatory disease. We will prescribe a drug, colchicine, and how your respond will be key to confirming the diagnosis.”*

*I tried to get up from where I was lying. I had never heard of FMF before. I had no idea how serious it was*.

*But one thing caught my attention as a mental health professional. According to my clinical observation about the language used in interpersonal relationships in my culture, there is always a problem if the subject of the sentence is “I” but is suddenly followed by “we.” For instance, a physician might say, “I am the doctor in charge of your treatment. I saw your test results; it seems that you have X cancer. We will try to do our best for your treatment.” Is the doctor referring to the healthcare team when he says “we”? Or is it simply because of others’ bad experiences, or because the possible outcomes are hard to talk about and “we” need support, even in a sentence? What was my doctor really saying? “We’ll need to do genetic testing.… We will prescribe a drug.” Such language indicates that something serious is on the way.*

*I felt weak and vulnerable. I was staggered to hear what he had said. They helped me, and I sat down. I turned my ear slightly in their direction so I could hear better, squinted my eyes, and grimaced.*

*“What did you say, sir? FMF? Is it chronic and hereditary? How so?”*

*He gave me information about the disease. He asked me to go to the hospital if the symptoms returned. Colchicine was prescribed, and I was discharged. Days later, when the genetic testing confirmed FMF, the question mark used to indicate a suspected diagnosis in patient records was removed. My questions had only just begun.*

## Discovery of Spiritual Meaning Through FMF

### Omnipotent Me

*I pretended as if I had never experienced what had happened to me until the diagnosis was confirmed by both genetic testing and the positive response to colchicine. I was only 26 years old. “I have a chronic illness at this young age, huh?” I thought. “The doctors are probably wrong anyway.”*

*I was fit and active. I got back to my daily routine*, *started to work hard, enjoyed my social life and my exercise, and tried to prove to myself how strong my body was. Besides, I was a soldier with duties to perform. I didn’t even look up what FMF was. I didn’t talk with anyone about my illness; I was glossing over the questions asked about my health.*

*I didn’t ask my family whether they had genetic backgrounds. How could they? Like me, my father was a soldier; he was strong like a rock, and he had no symptoms. Neither did my mother. Were they carriers of mutations in the FMF gene?*

*These thoughts—and many more—crossed my mind. I needed some answers. I called my mother and learned that FMF was also present in my relatives. The following day, I was holding the genetic test results, showing MEFV gene mutations. I couldn’t hold back my tears.*

While writing an autoethnography, a researcher selectively focuses on epiphanies. Epiphanies are the memories that significantly impact the trajectory of an individual’s life and that result from or are made possible by belonging to a culture and/or having a certain cultural identity (Hoque et al., 2017). Such epiphanies have an important role in one’s “self-concept.”

According to social identity theory, a self-concept is the unification of personal identity and social identity (Morris & Webb, 2022). While personal identity features personality traits and other characteristics that make each individual unique, social identity involves the groups that individuals belong to, including their community, religion, occupation, gender, and numerous others. Individuals tend to differentiate themselves by imagining what others think of them and then incorporating those perceptions into their self-concept (Reimer et al., 2020).

In my country, officers are selected for the army when they can meet the high standard of health, personal fitness, and toughness required for the profession, among many other qualifications. The same qualifications were needed to be a paratrooper and a diver. Moreover, my community regarded female army officers as a reputable professional position associated with power, health, and toughness.

Until the moment of diagnosis, my self-concept was likewise related to being powerful, healthy, and tough. Moreover, I thought I was invincible and omnipotent. But with FMF, my world flipped upside down. I felt that my life would never be the same. That is why I denied having the FMF diagnosis: it threatened my “omnipotent me” self-concept and the power that I believed I had.

Later, I realized that this feeling was related to feelings of despair and inadequacy. These were feelings I had never experienced in my life. At the time, I was not interested in any higher power or spiritual being on which I could call for support; after all, I had felt like a goddess my whole life, up until my diagnosis. Until then, I had always got what I wanted from life. At that moment, I realized that I have no power or control over my life. A goddess with no power. It was hard to accept.

When looking back from today’s perspective, I can see that my reactions at that time in my life coincided with grief reactions. Not only does the death of a person cause grief, but so does the loss of an ability, function, or health. In her book *On Death and Dying*, Elisabeth Kübler-Ross (1969) developed five stages of grief: denial, anger, bargaining, depression, and acceptance.

The “omnipotent me” phase seems to be consistent with denial, in which the individual responds to grief with avoidance, forgetting, procrastination, being easily distracted, acting mindlessly, keeping busy all the time, and thinking or saying, “I’m fine” or “it’s fine.” The denial phase includes specific concepts that align with the reactions described in my epiphany.

But still, I needed an approach that would further scientifically clarify my reluctance to talk about FMF. John Bowlby and Collin Murray Parkes propose the four phases of grief: shock and numbness, yearning and searching, disorganization and despair, and reorganization and recovery (Harris, 2019; Holmes & Holmes, 2014; Mikulincer & Shaver, 2022; Worden, 2018). To emotionally survive the initial shock of the loss, the grieving person feels numb and shuts down in the first phase of shock and numbness (Holmes & Holmes, 2014; Mikulincer & Shaver, 2022). Shut down defines my reaction: I was silent about what had happened to me. While Kübler-Ross developed her theory through the study of dying patients, it was based on the fatal aspect of the illness and reactions to it (Kübler-Ross, 1969). Bowlby and Parkes, meanwhile, focused their theory on attachment and loss of a loved one (Harris, 2019; Holmes & Holmes, 2014; Mikulincer & Shaver, 2022; Worden, 2018).

Still, these models were not directly involved with losing health and finding meaning. Much later, Kessler added a sixth stage to Ross’s model in his book *Finding Meaning: The Sixth Stage of Grief* (Kessler, 2019). In this newer model, finding meaning is the last stage of loss and grief.

In my experience, however, finding meaning is an ongoing process that starts with the first painful contact with the experience and never ends. In this context, Frankl’s (2004) concept of logotherapy is a useful framework. Logotherapy includes the meaning of human existence as well as humans’ search for that meaning (Frankl, 2004). All circumstances in life have meaning, and the meaning of life is not universal; it is unique for every individual, each of whom has a unique spiritual capacity to find that meaning. Individuals can find meaning by interacting with and appreciating their natural surroundings, by fully participating in loving relationships, and by experiencing suffering (de Carvalho & Moreira-Almeida, 2024; Frankl, 2004).

Yet neither the grief models nor logotherapy directly mentions the loss of the image of the “omnipotent me,” which for me has great importance in discovery of spiritual meaning. In my experience, this “omnipotent me” phase was the first stage of my grief, in which I became aware that humans are weak, not almighty, and have limited control in life. This stage was tied with the second phase: God’s punishment.

### God’s Punishment

*The days after the confirmed diagnosis were a nightmare. I searched all the resources I had access to. I had scientific papers in front of me, almost all of which reported negative outcomes from the disease. I was looking forward to hearing about patients’ experiences, but there was little to be found online.*

*For days, I passed by the rheumatology clinic without entering. Finally, I gathered my courage and started talking to patients. The things they told me were terrifying. One said FMF could be accompanied by other diseases; another mentioned kidney failure. “Also,” they said, “insomnia, fatigue, stress, carbohydrate-heavy diets, menstrual cycles, exercise, and other things can trigger episodes.” “What’s left?” I thought.*

*Everyone was talking about the risks and problems; no one was telling me how to deal with them. Doctors said that FMF is a chronic disease and that a complete cure is not possible, but that regular lifetime use of colchicine can prevent the onset of episodes. I should not even skip one dose. They said it was the only way I could live a normal life. “Use of medicine for a lifetime!” I thought. “Is that a normal life?”.*

*I was so upset, but not sad; I was just angry, angry against everyone and everything. I didn’t want to use any medication. I didn't want to be “the person with X illness.” I didn't know how to cope with a disease with unpredictable triggers at unpredictable times.*

*Moreover, everyone around me looked healthy and led a carefree life. Although I was a healthcare worker, I never assumed that I would have a chronic illness at this young age. Bad things always happened to others, not me. Where did I go wrong?*

*I had always been quite careful about the conditions that I could control, like my health, my sports, and, most of all, my diet. I couldn’t control my genes, too! I was deeply angry. I was angry not only about what I was experiencing but also about myself because I was not as strong as I thought. It felt entirely unfair.*

*Why me? I was a good person. I didn’t harm anyone. There are many bad people in this world. Did diseases happen to them? There must have been a mistake somewhere. It was like a punishment. If this was a punishment, what did I do to deserve it? If this was a punishment, who gave it to me? God?*

*I couldn't get out of my own head. I knew my thoughts weren’t reasonable, but I just couldn’t keep them quiet. Even though I was doing a PhD in mental health, I wasn’t even thinking about getting professional help. I didn’t want to talk about myself with anyone. I just needed to make sense of why this whole thing happened to me. I had dozens of questions but no answers. Furthermore, there was no one nearby who could give me these answers. I was desperate.*

An autoethnography should contain epiphanies that invite the reader to “relive” the experiences emotionally with the writer, allowing otherwise unspeakable experiences to be voiced (Richardson et al., 2005). Epiphanies should also provide ample details about facts and feelings and ensure the emotional credibility and vulnerability of the author by exposing pain, hurt, and betrayal, all of which make autoethnography compelling (Adams et al., 2021; Kennedy, 2020).

This phase of my experience was characterized by feelings of punishment, anger, weakness, and desperation, as well as questioning what had happened to me and why. I was trying to figure out what it all meant. The main feeling, I had deep inside was that I was facing a kind of punishment that I did not deserve. Who, then, was the punisher? Who has that power in my life? It seemed to me that the only one who could punish me must have been God.

In those days, I could not accept being closely connected to God. Maybe that was why I was being punished. It became crystal clear to me that I needed to connect with God, to accept that I am not almighty, but he is. The feelings of weakness and anger were freezing me, though; I did not know where to start.

Those reactions seem to be related to anger, the second stage of Kübler-Ross’s model. As described in her theory, at the time I was frustrated, pessimistic, irritable, sarcastic, aggressive or passive-aggressive, and out of control (Kübler-Ross, 1969).

In their model based on attachment theory and the loss of a loved one, Bowlby and Murray propose similar feelings in the second stage of grief, which they call yearning and searching (Harris, 2019; Holmes & Holmes, 2014; Mikulincer & Shaver, 2022; Worden, 2018). In this phase, sadness, anger, anxiety, and confusion are the major feelings, and individuals try to fill the emptiness created by the loss by knowing that the future imagined with what they have lost is no longer a possibility (Holmes & Holmes, 2014; Mikulincer & Shaver, 2022; Worden, 2018).

However, the dominant emotion in my epiphany was not only anger; I also felt punished by God, weak, and desperate. These feelings are emphasized in the third stage of their theory, disorganization and despair, in which the first acceptance of the reality of loss is experienced with feelings of hopelessness and despair, as well as anger and questioning. Bowlby and Parkes suggest that individuals in this stage often desire to withdraw and disengage from society and the activities they usually enjoy (Holmes & Holmes, 2014; Mikulincer & Shaver, 2022).

It seems that while both theories were consistent within themselves, neither was a perfect match with my experience of discovery of spiritual meaning. Thus, theological approaches may help in naming this step of the meaning-finding process by clarifying the meaning attributed to sickness (Tognacci, 2021).

In the ancient Hebrew tradition, sickness was caused by God’s wrath in response to human sins. According to the holy texts of Christianity, diseases are also connected to the wrath of God and come from weakness of faith, with the suggested remedies involving salvation through faith and love (McMartin & Hall, 2022; Tognacci, 2021). There appears to be some shared understanding among religions regarding the wrath of God. According to the moderate Islamic view, while people’s thoughts and actions cause their worldly troubles, it is Allah who creates these troubles, along with everything else (Sayilgan, 2023).

### God’s test

*I had to make new decisions and establish a new order for everything. As long car trips triggered my episodes, I had to travel by plane or stay and rest one night in a halfway hotel. Fatigue was another trigger for episodes, so I had to hire a housekeeper. Although these measures made my life a little easier, they posed a financial burden.*

*Sometimes I had to call my boss and get permission to rest at home because I was having episodes. My commanders were often supportive, but there were rules to obey. I had to take a note from my physician expressing that I needed to stay home from work and deliver it to my office for legal permission during those painful episodes. It was unbearable. For this reason, I prayed that my episodes would occur on days when I was not working. Sometimes, I felt compelled to go to work on the second or third day of an episode to resume my duties and not be a burden to my colleagues, who treated me with compassion and understanding.*

*The same was true for my family and social life. When important days such as wedding and funeral ceremonies coincided with the episodes, it was quite challenging for me. I felt trapped. I didn’t feel strong enough to handle everything at once. Meanwhile, I got married and got pregnant in the last year of my doctoral education. I wrote this in my diary:*

*“I stopped taking colchicine two months before I got pregnant. I am scared to put my baby at risk by using that Category C medicine! C means that studies in animals have revealed adverse effects on the fetus and that there are no controlled studies in women, or studies in women and animals are not available. Drugs from this class can be given to pregnant women if the benefit to the mother outweighs the risk to the fetus. I will never use it! How could I take such a risk with my baby?*

*In the 24th week of my pregnancy, I was told that there were week inconsistencies in fetal measurements; the gestational week was not compatible with head circumference or femur length. Doctors offered an amniocentesis to understand if there was a problem. Even amniocentesis might increase the risk of miscarrying the baby. What would I do if I learned my baby had a health problem? He is still my baby boy in every condition. I will not risk my baby, no, never, whatever it costs.”*

Remembering those days, I felt sorry again. I was always scared to be one of the 30% of infertile women who had ovulatory dysfunction and peritoneal adhesions because of FMF. In Turkish culture, having a child is a source of privilege and prestige in the community and is valued economically, psychologically, and socially. Infertility is primarily perceived as an embarrassing inadequacy of women, resulting in social stigmatization (Höbek Akarsu & Kızılkaya Beji, 2021). Thanks to Allah, I was pregnant. Amniocentesis was offered to understand if there was a problem with my baby. What would the results change?

In the Muslim community, it is believed that there is no doubt that Allah, the Exalted, is the only one who can give life and cause death. So, nothing would change for me even if he had health problems; I would surely give birth, and I would not terminate my pregnancy. Of course, I knew these were ethical issues for Muslims all around the world (Ekmekci, 2017).

Looking back now, I am aware that I was very pessimistic and always had the worst scenario in my mind. My non-adherence to treatment was one of the results of this attitude. Supposedly, I was trying to protect my baby from any risks, still forgetting that taking precautions does not prevent God’s will. I was overthinking things; I was desperate. I had a strong urge to take refuge in Allah, whom I realized I did not know very well.

As a part of the culture I was born into, I was also a natural part of the Muslim faith. However, I had picked up almost none of the holy books except out of intellectual curiosity. Maybe if I got to know Allah better, I could discover whether the things I had been living through were a punishment or not—and if they were, I could know what to do so this spiritual pain of mine would end.

I started reading the translation of the Holy Qur’an regularly, line by line, every day. I was hoping to find a single sentence to answer my question. At the very beginning of the Holy Qur’an, I met this verse: “We will certainly test you with a touch of fear and famine and loss of property, life, and crops. Give good news to those who patiently endure” (Qur’an, Surah Al-Baqarah, 2:155).

All of a sudden, I felt enlightened. It was not a punishment. It was a test about my life. Moreover, I was not alone. Everyone would “certainly” be tested with what I had been tested. What a relief. In his classic work *Theory and Practice of Group Psychotherapy*, Irvin Yalom (1995) identified 11 primary therapeutic factors in group therapies, one of which is called universality. Universality is the recognition of a shared experience and the knowledge that an individual’s problem is not unique. The sense that one is neither alone nor unique in one’s struggles and “the disconfirmation of the client’s feelings of uniqueness is a powerful source of relief” (Yalom & Leszcz, 2005). Universality is the act of getting people to connect on an emotional level, even without having the same experiences as each other (Yalom, 1995).

All my anxiety just flew away, and the feeling of “injustice” left me. Now I knew the answer. I was being tested, as everyone would be. That meant that I was also the one to find the right answer to that test. The answer was also given in the verse: I will have good news if I endure. Okay.

But how might one endure? What would I do to endure? I kept reading. “Who, when faced with a disaster, say, Surely, to Allah we belong, and to Him we will ˹all˺ return. They are the ones who will receive Allah’s blessings and mercy. And it is they who are ˹rightly˺ guided” (Qur’an, Surah Al-Baqarah, 2:156–157). That was what I needed—to receive Allah’s blessings and mercy.

The family, social, and occupational groups I belong to have always made me feel strong and supported. This time, however, my sense of belonging was bigger than everything else. I belonged to Allah, who owns and comprehends all things. I prayed for days, and then I kept reading: “When My servants ask you ˹O Prophet˺ about Me: I am truly near. I respond to one’s prayer when they call upon Me. So, let them respond ˹with obedience˺ to Me and believe in Me, perhaps they will be guided ˹to the Right Way˺” (Qur’an, Surah Al-Baqarah, 2:186). I felt I was not alone; I would be guided. I felt a strong sense of faith and surrender in my heart toward Allah, and I started to cry.

This was a subjective inner journey, of course. In contrast to traditional research methods, in which the individual with the chronic illness is invisible even as stories of the disease are told, epiphanies in autoethnography invite individuality back into human stories, valuing the individual experience (Richards, 2008). Writing an epiphany can be accepted as a way of knowing and a form of inquiry, allowing the individual experiencing the illness to make sense of his or her life (Adams et al., 2017; Hoque et al., 2017). Van Manen (2023) highlights writing as a method to verbalize inner speech or thoughts in which the writer must withdraw from the world to an inner space inside the self, as it is in this “solitary experience” that insight and depth of meaning can be written about.

According to Kübler-Ross’s theory, my experience at this period aligned with the bargaining stage, where the individual is occupied with overthinking and worrying, predicting the future and assuming the worst, and having feelings of guilt, blame, fear, anxiety, and insecurity. In that stage, she suggests that individuals often “negotiate with God” to live until an important date or occasion like their daughter’s wedding (Kübler-Ross, 1969). In my epiphany, I had those feelings that Kübler-Ross mentions, but it was not a matter of living or bargaining; it was more of an expectation that if I could get to know Allah better, my spiritual pain would end. In any case, it was clear that I was starting to recover spiritually.

Reorganization and recovery are the fourth phases of grief described by Bowlby and Parkes (Harris, 2019; Holmes & Holmes, 2014; Mikulincer & Shaver, 2022; Worden, 2018). According to this theory, individuals begin to return to a new state of normal, rebuild, and establish new goals and patterns of day-to-day life, realizing that life can still be positive, even after the loss. Intense feelings such as sadness, anger, and despair begin to diminish, and individuals’ faith in life starts to be restored. In my case, I can’t say that I completely recovered; I was still struggling, but my faith in Allah began to heal me.

### God’s mercy

*There was nothing I or anyone else could do about my baby anymore. I continued to do the things that made me feel at ease. I increased the frequency of my prayers. It was very good for me psychologically to seek refuge in the existence of a Creator who has absolute power in matters that I could not influence. As it is written in the Holy Qur’an, I had no choice but to surrender to the Creator, who created everything and has power over everything. I accepted my weakness; feelings of despair left me, and I felt peaceful instead. I felt that I had connected with Allah.*

*I wrote the following in my diary:*

*“In the Holy Qur’an, I realized how much he wrote about his compassion. The first surah starts with the basmallah: “In the name of Allah, the Gracious [Rahman], the Merciful [Raheem].” These were the two concepts that Allah chose to introduce himself with in the very first sentence. In general, it is recommended to say “Basmallah” before reciting any surah. So, whenever I cite the Qur’an, I always start with these two words showing his mercy.*

*“Raheem” also means womb or uterus in my mother tongue. I put my hand on my pregnant belly and said, “My God, when I had episodes, I had pain around here; when I am pregnant, I feel my baby kick around here. And now, when I put my hand here, I feel the warmth of your existence and mercy.”*

That’s when I realized that my illness was not a matter of punishment because Allah was Gracious and Merciful. It was not a matter of testing because the Qur’an was filled with many examples of human experience which may be considered as challenging tests, examples that clearly showed the virtuous actions that individuals should take (Pakravan & Motaharipour, 2019). How could it be a test when the answers were given? It was quite clearly an example of the “mercy of Allah”: He invited me to know him and connect with him.

After taking this journey, which started with my very first symptom of FMF, I do not feel omnipotent anymore. Instead, I feel surrendered to Allah, and I no longer need to have power. I don’t need a sense of control over every life event. Such a mindset was exhausting; now I feel free. I am not sad, I am not angry, and I do not feel anxious.

This journey completely transformed me. Now I know that all I have been living through was meant for this transformation. As it is written, “It may well be that you hate a thing while it is good for you; and it may well be that you love a thing while it is bad for you; and God knows, whereas you do not know” (Qur’an, Surah Al-Baqarah, 2:216). For me today, this perspective is like a lantern that lights my way.

By the time I earned my PhD, I had given birth. During my contractions, my baby’s arrhythmia started, so he was born by Cesarean section. After the C-section, I had an FMF episode with severe pulmonary involvement that affected my breathing. Regardless, on that day and in the years that followed, my son did not have any health problems rooted in my pregnancy or inconsistencies in fetal measurements.

This phase of my experience may be classified under acceptance, the fifth stage of Kübler-Ross’s (1969) model. In this theory, the stage of depression is mentioned before acceptance. Although I experienced short durations of low energy or deep sadness from time to time during this process, I can’t say that I experienced depression. In contrast, I felt hopeful before the acceptance stage. Kübler-Ross explains that an individual feels self-compassion, wisdom, validation, and courage in this stage by engaging with reality as it is, tolerating emotions, being present in the moment, adapting, coping, and doing so skillfully (Kübler-Ross, 1969).

Bowlby and Parkes proposed the fourth phase of grief as reorganization and recovery, suggesting that individuals become aware that their lives have changed and evolved into a new normal by understanding the positive aspects of their lives after loss (Harris, 2019; Holmes & Holmes, 2014; Mikulincer & Shaver, 2022; Worden, 2018). While both theories suggest nearly the same feelings I experienced, neither touches on discovery of spiritual meaning. My epiphany reflects how I lived a spiritual experience.

Spiritual experience is commonly defined in the literature as enlightenment, purpose, meaning, a sense of wholeness, a connection to something transpersonal, or an epiphany (Ayhan & Seki Öz, 2022; Kütmeç Yilmaz & Kara, 2021; Wahab, 2022). Doğan et al. (2020) mention that recovery does not only mean clinical recovery or personal recovery; it also refers to mental recovery, which is not only an outcome but also an ongoing process in which the individual and her new experience of the illness are comprehensively addressed. Mental recovery has related concepts such as individuality, support, relationships, hope, empowerment, and finding meaning (Doğan et al., 2020). They also suggest that finding meaning is only possible when individuals reevaluate their life, set new goals and take action, and realize the spiritual aspect of their experience (Doğan et al., 2020). There is no doubt that spiritual experience is affected by an individual’s religious context and upbringing (Asadzandi, 2020; Daher-Nashif et al., 2021).

This analytic autoethnographic study revealed four stages of discovery of spiritual meaning through FMF: “omnipotent me,” “God’s punishment”, “God’s test”, and “God’s mercy”. There are many quantitative studies and some qualitative studies on this topic, sharing various aspects of the search for spiritual meaning in Muslim culture. Systematic review and thematic analysis are powerful knowledge resources that ensure the full picture of the literature is visible (Moosapour et al., 2021; Saunders et al., 2023). The results of this autoethnographic study are discussed with the results of a systematic review and thematic analysis by Abdullah et al. (2020) conducted in Muslim patients and/or families for end-of-life care in Muslim-majority countries. They identified three themes and subthemes: selflessness (burden to others and caregiver responsibilities), ambivalence (hope and hopelessness), and strong beliefs in Islam (beliefs in death and afterlife and closeness to Allah) (Abdullah et al., 2020).

For “burden to others” subtheme, it is shown that patients had to maintain their strength in front of their family members and had the feeling of living only for them, particularly for their children. Patients reported lack of time, inability to carry out their daily household responsibilities, and experiences of symptoms affecting their lives and restricting their activities. In the subtheme “Caregivers’ Responsibilities” families are cited as a source of emotional support and motivation (Abdullah et al., 2020). These results support the findings I shared as my epiphanies about trying to maintain my self-concept as strong and control life by accepting myself “omnipotent”. Expectation of continuing role performance despite painful symptoms and making decisions that benefit not for my health but for my son is also appears to be common in both researches. As I mentioned in the “God’s Test” section, the family, social, and occupational groups I belong to have always made me feel strong and supported, as mentioned in “Caregivers’ Responsibilities” subtheme.

For the subtheme “hope and hopeless,” it is shown that facing terminal illness, patients reported being afraid and insecure. Extreme shock and hopelessness were reported on receiving diagnosis of cancer, leading to uncertainty. Nonetheless, they were still hopeful and positive about life. The motivation came from family support and Islamic religious belief that miracles can happen and one should not lose hope for a possible cure (Abdullah et al., 2020). These results are also quite similar to the findings of my study about “God’s punishment” in terms of feeling insecure, experiencing extreme shock, despair and hopelessness in the face of a chronic illness. Hope was also a subject in my epiphanies as “if I got to know Allah better, maybe I could discover whether the things I had been living through were a punishment or not—and if they were, I could know what to do so this spiritual pain of mine would end”. This viewpoint was the reason why I regularly read translation of the Holy Qur’an and met the verse “Give good news to those who patiently endure” (Qur’an, Surah Al-Baqarah, 2:155) which also gives hope miracles could happen.

The subtheme “Beliefs in death and afterlife” mentions that caregivers believed that their loved ones would be in heaven in the afterlife. In Islam, illness was considered a divine test from God and was viewed as taghdir (attitude to fate). Patients and family members reported accepting the diagnosis owing to beliefs that journey and destiny are part of plans by Allah (Abdullah et al., 2020). These results support my findings in “God’s Test,” where I wanted to understand the question and find an answer. It also supports my epiphanies in “God’s mercy” which is the answer. This illness was certainly part of the destiny for me to transform into a new person, as I wrote in my epiphany “after taking this journey, which started with my very first symptom of FMF, I do not feel omnipotent anymore. Instead, I feel surrendered to Allah, and I no longer need to have power,” and “this journey completely transformed me. Now I know that all I have been living through was meant for this transformation.”

The subtheme “Closeness to Allah” mentions that patients and caregivers reported adopting coping strategies by performing Islamic rituals: praying to Allah, reading the Qur’an, and engaging in other religious practices. These activities brought hope, peace and comfort. With disease, patients and caregivers reported to be closer to God and perform prayers in many situations; including while experiencing pain (Abdullah et al., 2020). My epiphanies mainly focus on how strongly I feel the existence of Allah’s mercy, even in my FMF episodes or when I have problems with my pregnancy. I also prayed to find hope, peace and comfort as mentioned in my epiphany: “*There was nothing I or anyone else could do about my baby anymore. I continued to do the things that made me feel at ease. I increased the frequency of my prayers. It was very good for me psychologically to seek refuge in the existence of a Creator who has absolute power in matters that I could not influence. As it is written in the Holy Qur’an, I had no choice but to surrender to the Creator, who created everything and has power over everything. I accepted my weakness; feelings of despair left me and I felt peaceful instead. I felt that I had connected with Allah.”*

### Limitations

This autoethnography has the limitation of a single narrative analysis in its generalizability, yet it has benefits to being able to achieve the depth of understanding of a chronic illness and the discovery of spiritual meaning and to evoke conceptual insights and to become a stimulus for further intellectual discussion for readers.

## Conclusion

Just as biological illnesses have psychological, sociological, cultural and spiritual effects, the human being should be viewed as a whole with these five characteristics. As a patient, in my long journey with FMF, I have experienced all these effects in my life that have transformed me to a new strong person who can use spiritual coping. There were sunny days and dark nights in this journey, which could easily be handled and managed carefully by professionals- if noticed. I recommend that researchers working in the field of mental health collaborate with rheumatologists and spiritual experts. I also recommend that FMF patients seek help in managing the biological, psychological, sociological, cultural and spiritual effects of the illness. Looking back, I realize that despite all the things I’ve been through regarding my mental health, I was unable to evaluate myself as a mental health professional in that context.

In this article I tried to invite readers to see FMF through my eyes. For me, FMF is no longer an illness; it is a unique part of my life, an experience that gave me a spiritual perspective to interpret myself, the world, others and the future. My process of finding meaning through my illness has been a spiritual journey. It may be beneficial to provide guidance to patients in this regard or to point them to resources where they can get guidance. I would like to share one more verse with the readers of this article who are having a hard time in their lives: “By the morning brightness, and [by] the night when it covers with darkness, our Lord has not taken leave of you, nor has He detested [you]. And the Hereafter is better for you than the first [life]. And your Lord is going to give you, and you will be satisfied. (Qur’an, Surah Ad-Duhaa, 93:1–5).

I am grateful to everyone who accompanied me though this experience—my parents, my family, my doctors and nurses, and my fellow FMF patients and readers of this article.
